# Thymus cyst: an option in the differential diagnosis of cervical-mediastinal tumors

**DOI:** 10.1590/S1808-86942010000400021

**Published:** 2015-10-19

**Authors:** Adriana Cartafina Perez-Bóscollo, Luciane Carneiro de Carvalho, Hebert Henrique Capuci, Marcelo C Fatureto, Sheila Jorge Adad

**Affiliations:** a Doctoral degree, physicians, professor; b Physician; c Physician; d Doctoral degree, physician and professor; e Doctoral degree, physician and professor

**Keywords:** thymic cyst, children, stridor

## INTRODUCTION

Congenital cystic and mediastinal neck masses include lymphangiomas, teratomas, neuroenteric cysts, thyroglossal duct cysts, branchial cleft cysts, vascular malformations and pulmonary hernias.^1^ These may be found at any level of the normal thymic descensus from the angle of the mandible to the upper mediastinum.^2^ Thymic cysts, which are rare, generally do not become included in the differential diagnosis, and therefore are rarely diagnosed preoperatively. Most of these cysts are asymptomatic, but about 6% may cause dysphagia, dyspnea, neck pain, stridor or rhonchus, which relate to its mediastinal extension.

## CASE REPORT

A boy aged 9 years was referred for an assessment of a left asymptomatic cervical mass that grew steadily with no inflammation during about 1 year. The physical examination detected a mobile tumor of fibroelastic consistency on the anterior left neck, which was difficult to palpate.

**Figure 1:**
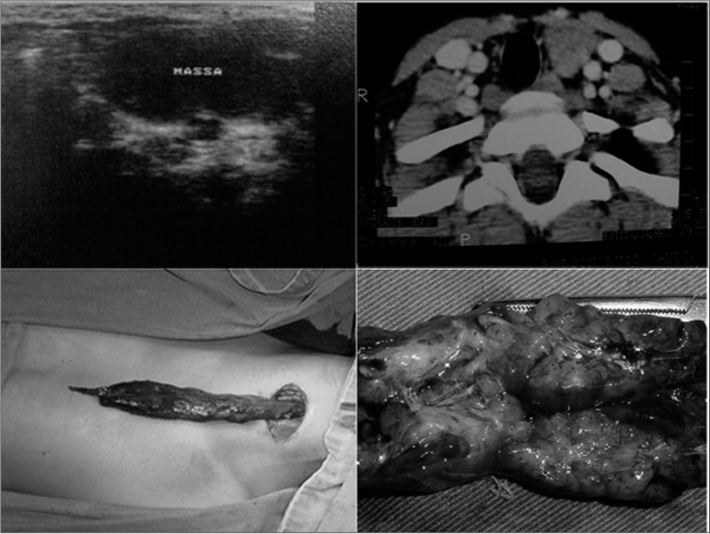
Ultrasound image of a cystic mass in the left cervical region, suggesting a branchial cyst; computed tomographic image showing a solid lesion in the parathyroid region, deviating the trachea to the right, suggesting a lymphoma. Cervicotomy showed a tumor extending into the mediastinum, which was resected uneventfully. Surgical specimen, a tumor sent for histopathology. A thymic cyst.

The mass did not hinder movements of the child. Ultrasound revealed a cystic mass with a possible diagnosis of a branchial cyst along the left jugulo-carotid chain with an inflammatory aspect; computed tomography showed a solid lesion in the left neck of unknown etiology, suggesting adenomegaly or a lymphoma. The mass extended towards the mediastinum. Left cervicotomy was undertaken for removal of the cervico-thoracic tumor; it had a benign appearance, glandular consistency, no necrotic areas, and extended into the anterior thorax (Fig. 1). Histopathology demonstrated a thymus within normal limits, with involutive alterations and cystic area lined with foreign body granulomas (reminiscent of cholesterol or keratin crystals) and a parathyroid gland within normal limits. There were no postoperative intercurrences, no neurological deficits or other injuries.

## DISCUSSION

The peak incidence of thymic cysts, in the first decade of life, may be explained by the fact that thymic residues are larger in the years before puberty. Although thymic cyst is not a frequent diagnosis, the abovementioned male patient in the first decade of life with a left cervical tumor falls within the common features of reports in the literature. It was a cyst lined with foreign body granulomas similar to cholesterol or keratin crystals, which are pathognomonic of thymic cysts;^3^ there were no symptoms, as was the case of most patients in Chiba's report.^4^ Ultrasound, which raised doubts about the diagnosis, is an examiner-dependent method; care is therefore needed when indicating treatment for rare cases. Given its cystic nature, the hypothesis of a branchial cysts was raised. Computed tomography revealed a solid mass, which suggested a lymphoma.

Left supraclavicular masses remind us of the abdominal lymphatic drainage, which was within normal limits. The choice was made to remove the tumor in the surgical theater under general anesthesia, applying perioperative measures pertaining to total tumor removal.

## FINAL COMMENTS

Thymic cysts are a rare cause of neck masses, which should nevertheless be included in the differential diagnosis, especially in children.^5^ This lesion is rarely diagnosed preoperatively, and may easily be mistaken with other neck masses.^6^ Thymic cyst images and their anatomical landmarks are well seen in computed tomography or magnetic resonance imaging. Once diagnosed, the treatment of choice is surgery; the mass may be symptomatic and esthetically displeasing, and may require confirmation at pathology to differentiate it from neoplasms. In the preoperative phase, a mediastinal thymus should be confirmed to avoid eth risk of total thymectomy. The prognosis after full removal of this lesion is excellent; no cases of recurrence have been reported.
